# Blood Pressure Measurement Validation Off the Cuff? Comment on “A New Cuffless Device for Measuring Blood Pressure: A Real-Life Validation Study”

**DOI:** 10.2196/10089

**Published:** 2018-10-22

**Authors:** Noud van Helmond, Timothy B Plante, Jeffrey I Joseph

**Affiliations:** 1 Department of Anesthesiology Sidney Kimmel Medical College Thomas Jefferson University Philadelphia, PA United States; 2 Department of Medicine Larner College of Medicine University of Vermont Burlington, VT United States

**Keywords:** cuffless blood pressure monitor, blood pressure monitor validation, hypertension

We read with interest the validation study of the commercially available CheckMe blood pressure (BP) monitor by Schoot et al [1]. We agree with the authors that the development of cuffless BP devices may improve rates of out-of-office BP measurement for adults with hypertension and that the value of such devices depends on the devices’ accuracy. We feel there are aspects of their report where clarification might be helpful.

One of the reported strengths of the study is that it met European Society of Hypertension International Protocol (ESH-IP) validation guidelines [2]. However, some aspects of the study deviated from the ESH-IP, including obtaining of measurements in varying anatomical positions. Usually, validation is performed in the manufacturer’s recommended position of measurement, which is typically in the same position as calibration [3]. Additional information would be helpful to better understand the performance characteristics from the device itself and to assess for bias introduced from protocol deviations.

First, presenting correlation coefficients and scatterplots can help readers assess the relationship between measurements from the devices. Next, the authors present a relative difference in mean systolic BPs, which is near zero. Presenting mean absolute difference between devices would be more informative to the accuracy of the device [2]. A modified version of the ESH-IP’s validation table is presented not in the usual numerical categories. Calculating these categories, the CheckMe deviates from the reference device by ≤5 mmHg, ≤10 mmHg, and ≤15 mmHg for 16%, 62%, and 86% of the measures, respectively. These performance characteristics do not meet the threshold of passing ESH-IP’s first part of validation. Finally, contrary to the description in the manuscript, we note that the reference device used has not undergone independent validation and its accuracy is not known [4]. Thus, it is difficult to interpret the accuracy of CheckMe without understanding the performance characteristics of the reference device.

We recognize that it is not possible to adhere to the ESH-IP with novel cuffless BP-measuring devices, since the ESH-IP assumes that the tested monitor does not require user-specific calibration. In response to the ongoing interest in cuffless BP measurement the Institute of Electrical and Electronics Engineers (IEEE) released a guideline specifically for the validation of these devices [5]. Because validation of a cuffless BP monitor immediately after calibration at the same BP could artificially increase its perceived accuracy [3], the IEEE protocol includes validation measurements after changes in BP from the calibration measurement and validation measurements after a significant period since calibration.

In conclusion, we encourage the authors to present additional analyses and results to improve the understanding of the CheckMe’s performance, and to follow up with a formal IEEE-protocol validation study. In the present study, the device is reported to be a Conformité Européenne-approved category IIa category medical device compliant with directive 93/42/European Economic Community, but it did not meet that directive’s accuracy requirements (EN 1060-3). The CheckMe has United States Food & Drug Administration approval for measurements of heart rate, oxygen saturation, temperature, and activity, but not for measurement of systolic blood pressure [6]. As the device does not meet ESH-IP accuracy criteria for blood pressure in this study, we are concerned that it is available for sale directly to consumers and may place adults at undue harm.
